# Auditory assessment of patients with acute uncomplicated *Plasmodium falciparum *malaria treated with three-day mefloquine-artesunate on the north-western border of Thailand

**DOI:** 10.1186/1475-2875-7-233

**Published:** 2008-11-06

**Authors:** Verena I Carrara, Aung P Phyo, Paw Nwee, Ma Soe, Hsar Htoo, Jaruwan Arunkamomkiri, Pratap Singhasivanon, François Nosten

**Affiliations:** 1 Shoklo Malaria Research Unit, PO Box 46, Mae Sot 63110, Thailand; 2 Faculty of Tropical Medicine, Mahidol University, Bangkok, Thailand; 3 Centre for Vaccinology and Tropical Medicine, Churchill Hospital, Old Road OX 3 7LJ, Oxford, UK

## Abstract

**Background:**

The use of artemisinin derivatives has increased exponentially with the deployment of artemisinin combination therapy (ACT) in all malarious areas. They are highly effective and are considered safe, but in animal studies artemisinin derivatives produce neurotoxicity targeting mainly the auditory and vestibular pathways. The debate remains as to whether artemisinin derivatives induce similar toxicity in humans.

**Methods:**

This prospective study assessed the effects on auditory function of a standard 3-day oral dose of artesunate (4 mg/kg/day) combined with mefloquine (25 mg/kg) in patients with acute uncomplicated falciparum malaria treated at the Shoklo Malaria Research Unit, on the Thai-Burmese border. A complete auditory evaluation with tympanometry, audiometry and auditory brainstem responses (ABR) was performed before the first dose and seven days after initiation of the antimalarial treatment.

**Results:**

Complete auditory tests at day 0 (D0) and day 7 (D7) were obtained for 93 patients. Hearing loss (threshold > 25 dB) on admission was common (57%) and associated with age only. No patient had a threshold change exceeding 10 dB between D0 and D7 at any tested frequency. No patient showed a shift in Wave III peak latency of more than 0.30 msec between baseline and D7.

**Conclusion:**

Neither audiometric or the ABR tests showed clinical evidence of auditory toxicity seven days after receiving oral artesunate and mefloquine.

## Background

Artemisinin derivatives, mostly artesunate and artemether, have been widely used in China and South-East Asia for the past 15 years, and are now recommended in combination therapy, in all malarious areas to prevent further spread of resistance [1]. In Thailand, the National Malaria Programme has used artesunate in combination with mefloquine as first line treatment for uncomplicated falciparum malaria since 1995 along the Thai-Burmese border [2,3]. This regimen has proved to be highly effective against multiple-drug resistant parasite strains, and its cure rate has remained above 90% since its introduction [4,5].

Clinical studies including thousands of patients confirmed that artemisinin-based combination therapies were safe and well tolerated in patients [6,7]. However, in animal studies, artemisinin derivatives have been consistently associated with neuronal damage, particularly in areas of the brainstem involved in hearing and gait control [8-13]. Neuropathological lesions were seen after prolonged administration of oral, intra-muscular and parental artemisinin compounds, but were more frequent after intra-muscular injection of oil-based arteether and artemether, than after parental or oral administration of artesunate [14-16]; clinical manifestations following oral administration of artemisinin derivatives were seen only at high doses [12]. In 1997, retrospective studies in Thailand [17] and in Vietnam [18] have specifically investigated the potential adverse effect on brainstem function in patients having been exposed to artemisinins. Patients were, in both studies, compared to controls who had not been treated with an artemisinin and who were living in the same environment and they were matched by age and sex; audiology results were similar in both groups and no neurological anomalies were found. However, concerns about safety of the artemisinin-based combinations have been raised following a retrospective study in Mozambique published in 2004 [19]. The authors reported a mild hearing loss across all but the two lowest audible frequencies in a non-randomised retrospective study of 150 construction site workers who had received artemether-lumefantrine. Audiograms were routinely performed on induction and cessation of employment. Audiology results of patients having received artemether-lumefantrine were compared to those from employees unexposed to this drug combination and matched by age, gender, weight and race. A negative change between the two audiograms was systematically seen among those having received artemether-lumefantrine, varying from -6.50 dBL to – 0.07 dBL. To further explore this issue the auditory function of 68 subjects treated with artemether-lumefantrine within the previous five years, and 68 age and sex-matched controls were assessed by the Shoklo Malaria Research Unit (SMRU), between October 2004 and March 2005. This retrospective study failed to show any difference in auditory function between the two groups [20]. More recently, two prospective studies evaluated the potential audiotoxicity of a standard oral dose of artemether-lumefantrine; one in 15 healthy volunteers followed up 8 days after treatment [21] and one in Ethiopian patients followed up for a period of 90 days [22]; neither study found pathological changes in audiometric pure-tone thresholds or ABR peak latencies following artemether-lumefantrine.

The present study aimed to evaluate prospectively the potential effects of artesunate in combination with mefloquine on the auditory function of patients with acute uncomplicated falciparum malaria treated in one of the SMRU clinic for migrant workers along the Thai-Burmese border.

## Methods

### Study site and study population

The study took place in the SMRU clinic located in Wang Pha, a village bordering Burma. The village is in an agricultural area of low malaria endemicity. The SMRU clinic offers clinical care to a large migrant population mostly coming from adjacent Burmese villages. Patients who had a positive rapid diagnosis test (Paracheck-Pf^®^, Orchid Biomedical Systems, Goa, India) were eligible for the study provided that they gave fully informed consent. A malaria smear was then performed to confirm the diagnosis of uncomplicated falciparum malaria. Severely ill patients, and hyperparasitaemic patients (4% or more parasites per 1,000 red blood cells on a thin malaria smear) were excluded from further tests, as well as those without *Plasmodium falciparum *parasites detected on the malaria smear or with a parasite count lower than five parasites per 500 white blood cells.

A medical history was taken and all previous antimalarial treatments verified against medical records. Patients with a history of severe or cerebral malaria, head trauma, coma or unconsciousness episode, chronic ear pathology or chronic neurological disorder, and those having taken drugs such as mefloquine, loop diuretics, or aminoglycoside antibiotics in the previous two months were excluded. All subjects were then assessed for a concomitant illness, and those with acute upper respiratory infection were also excluded. Patients with fever received paracetamol prior to the auditory tests performed by trained examiners. This investigation was part of a series of studies on the potential toxicity of antimalarial drugs, approved by the Ethical Committees of Oxford University (OXTREC) and Mahidol University (Bangkok).

### Audiology tests

A locally made sound proof chamber was available in the field setting, and special care was taken to reduce ambient noise as much as possible. Temperature and ventilation was maintained at 25°C by air conditioning during working hours; it was routinely turned off during the audiometry and the ABR tests. Ambient noise measured at the level of the test subject's head was within the American National Standard for maximum ambient noise levels for audiometric test rooms (ANSI 3.1–199).

#### Otoscopy and tympanometry

Otoscopic examination was performed before testing. Patients with eardrum perforation, acute ear infection or severe scarring of the tympanic membrane were excluded. Tympanometry was done using a Madsen™, Zodiac 901 tympanometer. Patients with an abnormal tympanometry at the day of enrolment such as a "type B" (flat wave), "type C" (wave peak shifted to the left with a middle ear pressure (MEP) lower than – 150 daP), or a wave peak with oscillations, in one or both ears were excluded and further tests were not performed. The mobility of the tympanic membrane was evaluated by the amplitude of the compliance peak.

#### Audiometry

The audiometer used was a Madsen™, Orbiter 922 desktop. Pure-tone air conduction thresholds were obtained for each ear separately using insert earphone to limit further the effect of ambient noise. The unmasked thresholds were established at 0.25, 0.5, 1, 2, 3, 4, 6 and 8 kHz using the modified Hughson-Westlake ascending procedure [23]. Subjects with abnormal hearing (pure-tone air conduction thresholds > 25 decibels (dB) at any tested frequency in either or both ears) were included in the study if the difference between the right and the left ear Wave V latency peaks was smaller than 0.20 msec. Patients with asymmetric hearing (a difference between ears of > 15 dB in pure-tone air conduction thresholds at three or more continuous frequencies), and those recalling a recent exposure to sustained loud noises and presenting with a pure-tone air conduction threshold of > 25 dB at frequencies of 2000 Hz and above were excluded from the study.

#### Auditory brainstem response (ABR)

The ABR test was performed using a portable computerized system (Bio-logic Navigator Pro AEP). Gold surface electrodes were applied to the vertex, both mastoids and the forehead. Patients rested on a wooden bed, and were allowed to relax before testing. Electrode impedance was checked before each test run and was maintained at < 5,000 Ohms for all electrodes. Impedance difference between electrodes was maintained at < 2,000 Ohms. A rarefaction click-stimulus was used to elicit the auditory evoked potentials. The duration of one click was 100 μs and the clicks were presented monoaurally at a rate of 11.1 per second with an intensity of 80 dB. A total of 2048 sweeps were recorded by the computer and results averaged. A contra lateral masking with 40 dB white noise was used. At least two replications were made to determine reliability. The procedure was performed for each ear separately. The waveforms were labeled I, III, V for the ipsilateral recording (tested-ear). The peak latency (PL) for each wave was established and the inter-peak latencies (IPL) (I to III, III to V and I to V) calculated automatically. Patients with inconclusive ABR tests (no reproducible or measurable waveforms), those with more than 10% artifacts during each run or for whom electrode impedance could not be maintained below requested values were excluded from the study.

### Treatment

All patients were treated with three days of artesunate (4 mg/kg/day), and mefloquine (25 mg/kg) given on the second (15 mg/kg) and third day (10 mg/kg) of treatment. Female patients of child bearing age had a pregnancy test prior to receiving antimalarial treatment. The first dose of treatment was received after all the auditory tests were performed and was supervised. If a patient vomited, a full dose was given again if the vomit was within half an hour of receiving the medication, and half a dose if it occurred between half an hour and an hour after. Patients unable to tolerate the drugs after three attempts had their medication changed and were excluded from the study. The two next doses were unsupervised, unless the patient had agreed to come daily to the clinic for the treatment, a practice commonly accepted in this setting where compliance is > 90%. No drug levels were done during the study.

### Follow up procedure

All patients who had completed the baseline (Day 0) auditory tests and tolerated their medication well were asked to come for a follow-up a week later (Day 7). A simple physical assessment was done, malaria smear was taken in patients with symptoms such as fever or headache, and the complete set of auditory tests was performed again.

### Statistical analysis

The primary endpoint of the study was based on ABR using Wave III, as it was expected to be the most sensitive parameter to detect any artemisinin derivative-induced toxicity [24,25]. A change from Day 0 in ABR Wave III peak latency in either or both ears of > 0.30 msec was considered as significant [26,27]. The range of pure-tone air conduction thresholds testing variability is usually within 5 dB; therefore, a threshold difference exceeding 10 dB between Day 0 and Day 7 was adopted as clinically significant.

If a patient had treatment failure, experienced a change in tympanometry, developed an ear infection, or had a significant change in the pure-tone air conduction thresholds at Day 7, the ABR results were not accepted.

Continuous normally distributed data were described by their mean and their standard deviation (SD), non-normally distributed data by their median, and range. Percentages were given for categorical data, which were compared using the Chi-square test with Yates' continuity correction or the Fisher's exact test. Paired t-test or Wilcoxon signed rank test were used to compare continuous variables. Demographic characteristics, treatment dose and clinical findings were analyzed in univariate analysis, or with Pearson's correlation, to evaluate possible factors associated with hearing loss at baseline, and changes in audiometry and ABR at Day 7.

All data were double entered using Microsoft Access, Version 2000 and analyzed using SPSS for Windows (SPSS Inc, Chicago, Illinois, USA), Version 11.0.

## Results

**Figure 1:**
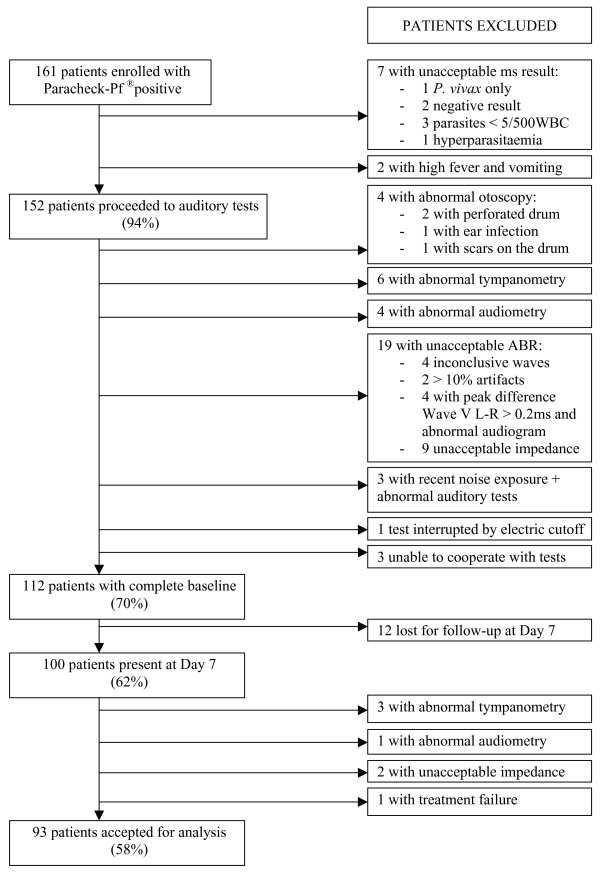
Patients flow diagram.

One hundred and sixty-one patients were enrolled in the study between June 2005 and December 2006, 49 were excluded and 12 lost for follow up (Figure 1). One hundred (62%) were followed at Day 7 and 93 (58%) completed the auditory tests and were entered in the final analysis. Their general characteristics are presented in Table 1. One patient was not clinically cured at Day 7 (1%); the malaria smear at Day 7 confirmed the presence of *P. falciparum *parasites.

**Table 1:** Baseline characteristics of the study population having completed Day 7 follow-up (n = 93)

**Patients' characteristics**	**All patients**
Male (n = 74)	79.6%
Age (in years)^a^	26.6 (8.8) [13–53], 13.7 – 41.2
Weight (in kg)^a^	50.7 (7.1) [30.0–70.0], 39.7 – 63.0
Tympanic temperature (in °C)^a^	37.2 (1.1) [34.9–40.1], 35.8 – 39.0
Mixed infection, PF+PV (n = 16)	17.2%
Geometric mean parasite count per μl [range]	7386 [79–190546]
Total artesunate dose (in mg)^b^	600 [300–825]
Previous artesunate treatment (n = 26)	28%
Time of last artesunate treatment (in month)^b^	4 [1 – 8]
**Tympanometry characteristics**	
Middle ear pressure, left ear (in daP)^b^	-15 [-145 – +25]
Middle ear pressure, right ear (in daP)^b^	-15 [-140 – +95]
	
Static compliance, left ear (in ml)^b^	0.48 [0.16–3.08]
Static compliance, right ear (in ml)^b^	0.46 [0.15–2.52]
	
Tympanometric gradient, left ear (dimensionless)^a^	0.49 (0.15) [0.19–0.83], 0.22 – 0.77
Tympanometric gradient, right ear (dimensionless)^a^	0.46 (0.15) [0.09–0.81], 0.23 – 0.74

^a ^Mean value, with (SD), [range], 5^th ^and 95^th ^percentiles without brackets

^b ^Median, [range]

### Tympanometry

Tympanometry was performed for all patients. Six patients with MEP values >-150 daP or with oscillations at baseline were excluded from the study. Two patients had an abnormal tympanometry at Day 7 (MEP >-150 daP); tympanometric values of the remaining patients were unchanged at Day 7 compared to baseline.

### Audiometry

Twelve patients were excluded after completion of the auditory tests: eleven on admission and one at Day 7 follow-up. Five of them had a threshold difference between left and right ear of more than 15 dB at three or more frequencies; four had at least one pure-tone air conduction threshold above 25 dB and a wave V inter-latency > 0.20 msec on ABR, and the three last patients with abnormal results reported a recent loud noise exposure.

**Figure 2:**
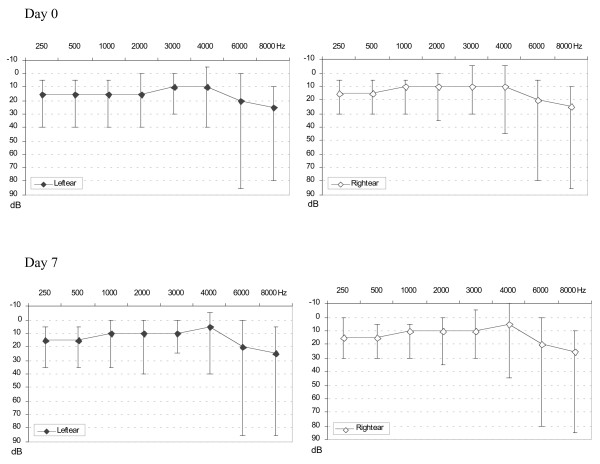
**Pure-air tone conduction thresholds at Day 0 and Day 7, both ears separately.** Air-conduction threshold is given as median values with [range], dBL.

Air-conduction threshold median values and their range by frequencies for the remaining patients are presented in Figure 2. Hearing loss (threshold > 25 dB) in at least one tested frequency in one or both ears was present among 57.0% (53/93) patients, ranging from 1 to 8 frequencies (median: 2 frequencies); hearing loss was bilateral in 33/53 patients (62%); when unilateral, right or left ears were equally affected. The hearing impairment involved high frequencies (6 and 8 kHz) in the majority of cases (44/53, 83%). Threshold values were between 30 and 40 dB for 45 of those patients (85%), equivalent to a mild hearing loss according to the American National Standards Institute (ANSI S3.6, 1996), between 45 and 70 dB for 7 (13%) or moderate hearing loss, and at 85 dB for 1 patient (severe hearing loss). Hearing loss was associated in univariate analysis to age (*P *< 0.001), but not to sex, body weight, temperature, parasitaemia, tympanometry results, or time to last artesunate treatment.

Nine of the 53 patients with one or more pure-tone air conduction thresholds above 25 dB at baseline returned to thresholds within normal limits at Day 7, and 12 had at least one tested frequency returning to a threshold ≤ 25 dB. Three patients had a unilateral change from 25 dB to 30 dB (2 patients) and from 25 to 35 dB (1 patient) at the highest frequency (8,000 Hz) between baseline and Day 7. Nobody had a threshold change exceeding 10 dB between Day 0 and Day 7 at any tested frequency. Overall, there was an improvement of 0.9 dB to 1.8 dB in all air-conduction thresholds at Day 7 on the left ear (statistically significant at all but 3,000 Hz frequency), and 0.4 to 1.5 dB on the right ear, statistically significant at all but 3 frequencies (3,000, 6,000 and 8,000 Hz). This improvement was correlated with the intensity of fever at baseline (*r *= 0.23, *P *< 0.001), but not with the level of parasitaemia (*r *= 0.026, *P *= 0.12). The total expected dose of artesunate or mefloquine received did not affect the hearing threshold changes, and neither did the severity of hearing loss at baseline.

### Auditory brainstem response

After completion of the ABR, nineteen patients were excluded at baseline and two at Day 7 of follow-up (Figure 1). Ninety-three paired ABR were analyzed. Mean peak latencies and interpeak latencies for both ears separately are presented in Table 2. There was a small but significant difference in Wave I peak latency between ears at baseline (mean value left ear 1.52 msec *vs*. 1.55 msec on the right, *P *= 0.006); this difference became non-significant at Day 7. No patient showed a shift in Wave III peak latency of more than 0.30 msec between baseline and Day 7. However, there was a small (within 1 SD of the baseline), but clear delay in peak Wave I–III and V latencies at Day 7 compared to baseline. Delay in peak Wave I–III and V latencies was present in 74% (69/93), 80% (74/93) and in 81% (75/93) of patients respectively. Peak Wave III and V latencies delay was more likely to be bilateral (62% *vs*. 38%, *P *= 0.005 and 64% *vs*. 36%, *P *= 0.001). Among those, 17 patients (29%) had a bilateral delay in all three peak Wave latencies and 19 others (33%) had a bilateral delay in peak Wave III and V. Patients with fever ≥ 37.5°C on admission were more likely to have a delay in Wave III and V peak latencies than those without fever (42% *vs*. 11%, *P *= 0.02 and 41% *vs*. 11%, *P *= 0.03 respectively) and this delay was more pronounced, 0.1 msec *vs*. 0.04 msec for Wave III and 0.1 msec *vs*. 0.05 msec for Wave V peak latencies, *P *= 0.002 and *P *= 0.003 respectively. Delayed peak latencies were not artesunate or mefloquine dose-dependant, and were not associated with sex or age of the patients, their parasitaemia on admission, tympanometry results, hearing loss or changes in the air conduction thresholds between admission and Day 7.

**Table 2:** Auditory Brainstem Responses (ABR) at Day 0 and Day 7, for both ears separately

	**Peak latencies**			**Inter-latencies**		
	**Wave I**	**Wave III**	**Wave V**	**I–III**	**III–V**	**I–V**
Day 0						
Left ear	1.52 (0.14)	3.67 (0.15)	5.45 (0.22)	2.15 (0.16)	1.78 (0.17)	3.93 (0.24)
Right ear	1.55 (0.13)	3.65 (0.14)	5.44 (0.23)	2.10 (0.16)	1.79 (0.17)	3.89 (0.25)
Day 7						
Left ear	1.55 (0.14)	3.73 (0.16)	5.51 (0.22)	2.18 (0.17)	1.78 (0.17)	3.96 (0.24)
Right ear	1.57 (0.13)	3.71 (0.14)	5.54 (0.23)	2.14 (0.17)	1.82 (0.17)	3.97 (0.25)
Difference D7-D0						
Left ear	0.03 (0.07)	0.07 (0.09)	0.07 (0.11)	0.04 (0.10)	0.00 (0.08)	0.04 (0.12)
*P *value	< 0.001	< 0.001	< 0.001	0.001	0.892	0.003
Right ear	0.02 (0.06)	0.07 (0.08)	0.09 (0.13)	0.05 (0.08)	0.03 (0.11)	0.07 (0.13)
*P *value	0.003	< 0.001	< 0.001	< 0.001	0.017	< 0.001

Presented as mean, (SD), units are in msec.

## Discussion

The artemisinin derivatives are essential in the treatment of uncomplicated falciparum malaria, and they are more effective than quinine in severe malaria [28]; their use worldwide has increased at an exponential rate as drug resistance spreads. Artemisinin compounds are considered safe and well tolerated [6,7]. The main concern about toxicity comes from experimental studies on animals. Those studies have consistently found a neurotoxic effect on certain brainstem nuclei and cerebellar roof nuclei involved in hearing and gait control [10,12]. This toxicity appears to be dose-dependant and varies according to the mode of administration; oil-based compounds such as artemether and arteether, administered intra-muscular, are more toxic than intravenous water-soluble artesunate or oral administration of any of the above substances [9,11,15,29,30].

This study is the first prospective study assessing the effects on auditory function of a standard oral dose of artesunate (4 mg/kg/day) combined with mefloquine (25 mg/kg) in patients with uncomplicated falciparum malaria. There was a small improvement at all tested frequencies in both ears seven days after initiation of treatment, correlated to the degree of fever on admission. This improvement was not substantial enough to be spontaneously reported by any of the patients and could also have been the result of a learning effect and a better concentration during the test after fever resolution. A similar improvement was found in the study of Gurkov et al. in patients treated with artemether-lumefantrine and followed up 90 days [22].

Three patients had a measurable reduction in hearing threshold (5 dB for 2 patients and 10 dB for the last one); however none complained of a hearing loss. Although those findings remain unexplained they seem unlikely to be due to an ototoxic drug effect (asymmetric hearing loss, at the highest frequency only). Overall our audiometry results are similar to the results reported by Mc Call et al. among 15 healthy volunteers who underwent experimental malaria and received artemether-lumefantrine [21].

ABR peak latencies and inter-peak latencies measures (the primary endpoint of this study) were similar at baseline to those reported by previous studies conducted along the Thai-Burmese border [17,20]. A minimal, but significant, prolongation of all Wave peak latencies was observed at Day 7, but not of the inter-peak latency III–V, which would have been prolonged in case of artemisinin toxicity, according to the results from the animal studies. There was no relationship between total dose of artesunate received, or of mefloquine, and Wave peaks delay. On the other hand, patients with fever on admission were more likely to have a prolongation of Wave peak latencies at Day 7 compared to baseline. Changes in body temperature affect the evoked potentials and an increase in temperature tends to reduce the peak latencies for all waves, in animals [31-33], during exercise [34], and in sick patients [35], a phenomenon which could explain the findings of this study.

The ABR test is a sensitive method to detect abnormalities along the auditory pathway, and was well tolerated even by sick patients. It failed to show a significant change in inter-peak latency III–V or any abnormally delayed Wave III peak latency after completion of the antimalarial treatment.

At baseline, a majority of the patients enrolled in the study presented with some hearing loss in the high frequencies (6–8 kHz) associated with age only. This finding remains unexplained, but does not appear to be related to prior exposure to artesunate. It could be due to a combination of factors such as aging, noise-exposure, prior use of antibiotics and smoking, all frequently found in this population, and all of which might be involved in high frequencies auditory deficit [36-40].

## Conclusion

The results of this prospective study are reassuring and do not support the hypothesis of a toxic effect of the artesunate or the mefloquine on the auditory pathway.

## Authors' contributions

VIC participated in the design and co-ordination of the study, the collection and analysis of data and drafted the manuscript. APP participated in the co-ordination of the study, the collection and analysis of data and helped to draft the manuscript. PN, MS, HH and JA recruited patients, performed the audiology tests and collected the data. PS and FN conceived of the study and participated in its design and helped to draft the manuscript. All authors read and approved the final manuscript.
